# Learning curve of ultrasound-guided percutaneous central venous port placement in children

**DOI:** 10.1186/s12887-024-04990-x

**Published:** 2024-08-07

**Authors:** Ho Jong Jeon, Kyong Ihn, In Geol Ho

**Affiliations:** 1 Division of Pediatric Surgery, Department of Surgery, National Health Insurance Service Ilsan Hospital, Goyang, Republic of Korea; 2 Division of Pediatric Surgery, Department of Surgery, Ludlow Faculty Building, Severance Children’s Hospital, Yonsei University College of Medicine, 50 Yonsei-Ro, Seodaemun-Gu, Seoul, 03722 Republic of Korea

**Keywords:** Central venous port, Learning curve, Complication rate, Operative time

## Abstract

**Background:**

Although percutaneous central venous port (CVP) placement can be quickly performed using minimally invasive surgery, short- and long-term complications can occur. Beginner pediatric surgeons must overcome learning curves influencing operative time and complication rates. However, few studies have been conducted on the learning curve of ultrasound-guided percutaneous CVP placement. This study analyzed the progress, results, complications, and learning curve of ultrasound-guided percutaneous CVP placement in children performed by a single beginner pediatric surgeon.

**Methods:**

Data from 30 children who underwent ultrasound-guided percutaneous CVP placement were reviewed. The patient characteristics, procedure indications, access veins, operator positions, operative times, and complication rates were analyzed.

**Results:**

Cumulative sum analysis revealed two stages in the learning curve: stage 1 (initial 15 cases) and stage 2 (subsequent cases). There was a correlation between the number of cases and operative time (Pearson correlation = -0.499, *p* = 0.005); the operative time was significantly longer in the first than in the second stage (*p* = 0.007). Although surgical complications occurred more frequently in the early (26.7%) than in the late stage, it was not significantly different between the two stages (*p* = 0.1). During the study period, the operative time was significantly reduced owing to the change in the operator’s position from the patient’s right side to the patient’s head (*p* = 0.005).

**Conclusions:**

Ultrasound-guided percutaneous CVP placement was a safe surgery that allowed a beginner pediatric surgeon to overcome the learning curve after only 15 cases and involved a relatively small number of complications compared with other pediatric surgeries. Additionally, the suitable position of the operator affected the surgical outcomes.

## Background

Central venous port (CVP) placement is necessary in pediatric patients diagnosed with malignancies requiring chemotherapy or chronic conditions requiring long-term intravenous treatment [1, 2]. Compared with tunneled central lines, implantable CVPs are associated with lower rates of bloodstream infection and a lower likelihood of requiring revision or replacement over the duration of therapy [3]. CVP placement surgery in children requires access to an experienced pediatric surgeon and general anesthesia due to the small size of the anatomical structures, and the fear of interventional radiological procedures under local anesthesia cannot be overcome as in adults.

CVP placement in children is a safe procedure with a low complication rate [4]. Previous studies have shown that ultrasonography-guided venous access is preferred for both adults and children, and many pediatric surgeons opt for this procedure for CVP placement [4–7]. Although CVP placement can be performed quickly using minimally invasive surgery, short- and long-term complications may occur, including pneumothorax, hemothorax, arterial puncture, infection, hematoma, thrombosis, and wound problems [8–10]. Pediatric surgeons will likely experience these complications at least once.

Beginner pediatric surgeons must overcome learning curves that influence both operative time and complication rates; however, few studies have been conducted on the learning curve of ultrasound-guided percutaneous CVP placement. This study aimed to analyze the progress, outcomes, complications, and learning curve of ultrasound-guided percutaneous CVP placement performed by a single beginner pediatric surgeon and share the results with other beginner pediatric surgeons.

## Methods

### Study design

This retrospective study included 30 patients who underwent ultrasound-guided percutaneous CVP placement by a single beginner pediatric surgeon at Severance Children’s Hospital between July 2021 and December 2022. The study was approved by the National Health Insurance Ilsan Hospital (No. 2023–03-046) and the Severance Hospital, Yonsei University Health System (No. 4–2022-1531) Institutional Review Board, and it was conducted according to the tenets of the Helsinki Declaration. This retrospective study was waived from informed consent by the National Health Insurance Ilsan Hospital and the Severance Hospital, Yonsei University Health System Institutional Review Board. Patient characteristics, procedure indications, access veins, operator positions, operative times, and complication rates were obtained from patients’ medical records and analyzed. The operative time was defined as the time from the operator’s declaration of the start of surgery to the completion of the operation site closure.

## Surgical procedure for ultrasound-guided percutaneous CVP placement

The product used in all CVP placements was the Powerport® (Bard Access Systems, Salt Lake City, UT, USA). CVP lines were inserted into the right or left internal jugular veins under general anesthesia using the ultrasound-guided Seldinger technique. Key steps include:

1. Vein Identification and Puncture: The internal jugular vein is identified using continuous ultrasound guidance and punctured with a 21-gauge needle.
2. Guidewire Insertion: A guidewire is inserted through the needle into the superior vena cava, followed by needle removal and site compression to prevent hematoma.
3. Incision and Pocket Creation: An incision matching the CVP diameter is made, the pectoralis major fascia is exposed, and a subcutaneous pocket is created to secure the CVP.
4. Securing the CVP: The CVP is fixed to the pectoralis major fascia with a nonabsorbable suture to prevent migration.
5. Catheter Pathway Preparation: A small incision parallel to the clavicle is made to allow the introduction of a tunneler, vessel dilator, and sheath introducer. A subcutaneous tunnel is created from the pocket to the puncture site.
6. Catheter Insertion: The catheter is cut to length, and its tip is placed at the junction of the superior vena cava and right atrium. The vessel dilator and guidewire are removed, and the CVP catheter is inserted through the sheath into the vessel.
7. Flow Test and Securing: The sheath is removed, and a flow test is conducted to ensure no obstructions or leaks. The catheter is flushed and locked with heparinized saline.
8. Verification: The correct position of the catheter tip is verified with portable chest radiography.

### Statistical analyses

Cumulative sum (CUSUM) analysis was used as a quantitative method to analyze the learning curve. The t-test, Chi-squared test, Fisher’s exact test, and Mann–Whitney U-test were used for the comparative analysis of each data point. Statistical significance was set at *p* < 0.05. All statistical analyses were performed using IBM SPSS Statistics ver. 26.0 (IBM Co., Armonk, NY, USA).

## Results

The cohort (*N* = 30) included 18 males (60.0%) and 12 females (40.0%). The median age at which surgery was performed was 6.7 (3.5–10.3) years. The patients’ median weight at the time of surgery was 23.8 kg (15.7–41.7), median height was 120.3 cm (99.3–142.3), and median body mass index (BMI) was 17.7 kg/m^2^ (15.7–20.2). The most common indication for surgery was oncologic disease (96.7%) (Table 1).

**Table 1:** Comparison of demographic data and treatment outcomes

	**Entire cohort (*n* = 30)**	**Stage 1 (*n* = 15)**	**Stage 2 (*n* = 15)**	***p*-value**
Sex				1.0
Male	18 (60.0%)	9 (60.0%)	9 (60.0%)	
Female	12 (40.0%)	6 (40.0%)	6 (40.0%)	
Age at operation (years)	6.7 (3.5–10.3)	5.5 (3.1–8.8)	9.2 (3.5–11.9)	0.174
Weight (kg)	23.8 (15.7–41.7)	20.6 (16.6–34.9)	40.7 (15.3–50.0)	0.325
Height (cm)	120.3 (99.3–142.3)	119.2 (98.3–135.0)	137.9 (100.0–146.0)	0.25
BMI (kg/m^2^)	17.7 (15.7–20.2)	17.3 (15.7–19.2)	19.0 (16.4–22.1)	0.217
Indications				1.0
Oncology^a^	29 (96.7%)	15 (100.0%)	14 (93.3)	
Others (general condition)^b^	1 (3.3%)	0 (0.0%)	1 (6.7%)	
Access veins				1.0
Right internal jugular vein	29 (96.7%)	15 (100.0%)	14 (93.3)	
Left internal jugular vein	1 (3.3%)	0 (0.0%)	1 (6.7%)	
Operator’s position				0.017
Patient’s right side	6 (20.0%)	6 (40.0%)	0 (0.0%)	
At the patient’s head	24 (80.0%)	9 (60.0%)	15 (100.0%)	
Operative time (min)	40.5 (24.0–16.0)	43.0 (40.0–50.0)	29.0 (20.0–43.0)	0.007
Complications	4 (13.3%)	4 (26.7%)	0 (0.0%)	0.1
Follow-up duration (days)	181.0 (53.0–257.5)	254.0 (208.0–296.0)	54.0 (43.0–117.0)	< 0.001

Data are presented as median (interquartile range) or number (%)

^a^Leukemia (11 cases, 37.9%), brain tumors (8 cases, 27.6%), solid tumors (8 cases, 27.6%), and lymphomas (2 cases, 6.9%)

^b^Mitochondrial cytopathy with pneumonia

**Fig. 1:**
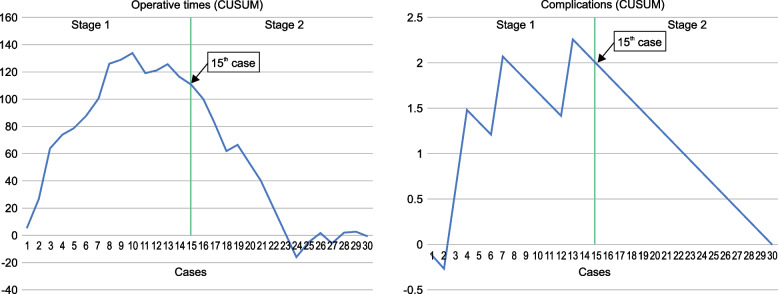
Cumulative sum (CUSUM) curves of operation times and complications for central venous port placement procedures

The chart of the CUSUM analysis, according to the total procedure time and number of complications, revealed two stages of the learning curve (Fig. 1). The CUSUM trend demonstrated an upward slope from the 1st to the 15th case and a sharp decline from the 16th case onward. Therefore, two stages were defined: stage 1, corresponding to the section from the 1st to the 15th case, and stage 2, corresponding to the section from the 16^th^ to the 30^th^ case.

Based on these results, a comparison of the demographic data and treatment outcomes is shown in Table 1. The median age at which surgery was performed showed no difference between the two stages (*p* = 0.174). Patients in the two stages did not differ regarding weight (*p* = 0.325), height (*p* = 0.25), or BMI (*p* = 0.217). The most common access vein was the right internal jugular vein (96.7%), with no significant difference between the two stages. The operator’s position changed during the study period (20.0% of procedures performed on the patient’s right side and 80.0% at the patient’s head); the difference between the two stages was statistically significant (*p* = 0.017). The median operative time was 40.5 min, significantly different between the two stages (*p* = 0.007).

Complications were more frequent in the early than late stage (26.7% vs. 0.0%, respectively). However, the difference was not statistically significant (*p* = 0.1). Complications occurred in four cases, including percutaneous puncture site hematoma, CVP catheter thrombosis, CVP site infection, and arrhythmia due to an excessively long catheter tip position. Although surgical complications occurred, there was no first failure during the surgical procedure, so there were no second or more attempts. The median follow-up duration was 181 (53.0–257.5) days.

**Fig. 2:**
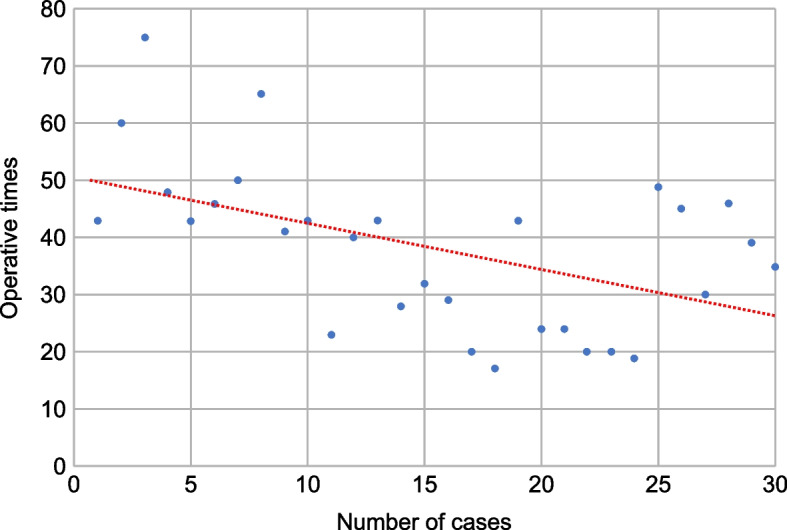
Correlation between the increasing number of cases and operative time. Pearson correlation = -0.499, *p*  = 0.005

The increasing number of cases correlated with the operative time (Pearson correlation (r) = -0.499, *p* = 0.005; Fig. 2). No significant correlations were found between the operative time and patient age (*r* = -0.193, *p* = 0.308), weight (*r* = -0.213, *p* = 0.258), height (*r* = -0.221, *p* = 0.240), or BMI (*r* = -0.099, *p* = 0.604).

The operator’s position started on the patient’s right side, as learned from a supervisory pediatric surgeon, but was moved near the patient’s head according to the operator’s preference. There was no significant difference between the operator’s position and the occurrence of complications. However, the operative time was significantly reduced owing to the change in the operator’s position (*p* = 0.005; Table 2).

**Table 2:** Comparison of procedure outcomes according to the operator’s position

	**Patient’s right side (*n* = 6)**	**At the patient’s head (*n* = 24)**	***p*-value**
Operative time (min)	47.0 (43.0–63.8)	33.5 (23.3–43.0)	0.005
Complications	2 (33.3%)	2 (7.7%)	0.169

Data are presented as median (interquartile range) or number (%)

## Discussion

General approaches to central venous catheters include traditional open surgical venous cutdowns and percutaneous procedures [7, 11–17]. Ultrasound-guided percutaneous central venous catheter placement was first described in adults in the 1990s and is widely used in all age groups, including children [13–17]. Large-scale studies on ultrasound-guided percutaneous CVP placement in children, as well as studies on the learning curves associated with various central venous catheter placement procedures in children, have been published [4, 16, 17]. However, few studies have been conducted on the learning curve of ultrasound-guided percutaneous CVP placement.

Using CUSUM analysis, this rare study on the learning curve of ultrasound-guided CVP placement in children demonstrated the presence of two learning stages: before and after completing procedures in 15 cases. There was a statistically significant relationship between the number of CVP placements performed and operative time: as the number of cases increased, the operative time decreased. Although the statistical values differ, our study’s median operative time of 29 min, compared with the mean value of 40 min in another study, is an advanced surgical outcome [4]. Previous studies have reported that 8–20 cases of ultrasound-guided percutaneous central venous catheter placement are sufficient for training under the supervision of an experienced surgeon [16, 18]. In this study, although additional port-placement techniques were added, 15 cases were required to achieve a learning effect.

According to the literature, the complication rates of ultrasound-guided percutaneous central venous catheter placement in children vary between 2.4% and 13.9% [4, 19–21]. The complication rates in our study tended to be higher than those reported in the literature, likely owing to various factors; first, this study was conducted in a relatively small number of patients over a short research period. Second, as a study of the learning curve of a beginner pediatric surgeon, differences in proficiency are also a likely factor. There was no significant difference in the complication rates between the two stages of the CUSUM analysis learning curve; this can also be attributed to the aforementioned factors. Among the four patients with complications, three cases — including CVP catheter thrombosis, CVP site infection, and arrhythmia due to a catheter tip that was too long — required a CVP change. No major life-threatening complications were observed.

This study demonstrated a relationship between the operator’s position and surgical outcome, which was not previously investigated. In the first six cases, the operator’s position was on the patient’s right side, which was learned from the supervisory pediatric surgeon. However, depending on the surgeon’s preference, the procedure was moved near the patient’s head, and the remaining 24 cases were performed as such. In ultrasound-guided percutaneous CVP catheter placement, the operator’s position is not rigidly established; rather, it is determined by the operator’s habits and preferences. The supervisory pediatric surgeon performed the procedure on the patient’s right side based on his preference and to reduce position changes and operative time. The beginner pediatric surgeon initially proceeded accordingly. However, after the beginner pediatric surgeon experienced a complication of percutaneous puncture site hematoma due to discomfort during the procedure, the operator position was moved near the patient’s head due to preference. Although there was no relationship between the operator’s position change and complication rate, the reduction in the operative time was a statistically significant outcome. Thus, a suitable operator position affected the surgical outcome owing to learning and experience.

This study has a few limitations, as it is a short-term retrospective study of only one beginner pediatric surgeon with a small number of patients. It is expected that statistically significant results regarding complication rates will be obtained through long-term research involving more patients and more beginner pediatric surgeons.

## Conclusions

Using a CUSUM analysis, the current study revealed two stages in the learning curve of ultrasound-guided percutaneous CVP placement in children — before and after 15 cases — and demonstrated improved outcomes regarding operative times. Additionally, a suitable operator’s position affected the surgical outcomes.

Nevertheless, ultrasound-guided percutaneous CVP placement is considered a safe surgery that allows a beginner pediatric surgeon to overcome the learning curve with only 15 cases and involves a relatively small number of complications compared with other pediatric surgeries. Overcoming the learning curve of ultrasound-guided percutaneous CVP placement is essential for reducing the operative times and complication rates.

## Data Availability

Most of the data generated or analyzed during this study are included in this article. Limited data can be provided on request to the main author (Dr. Ho Jong Jeon) at gdijhj@nhimc.or.kr.

## Abbreviations

BMI: Body mass index
CUSUM: Cumulative sum
CVP: Central venous port
